# Nonatopic Eosinophilic Esophagitis in an Adult

**DOI:** 10.7759/cureus.31967

**Published:** 2022-11-28

**Authors:** Ivanna Ward, Davong D Phrathep, Kevin D Healey, Stefan Anthony, Michael Herman

**Affiliations:** 1 Medicine, Philadelphia College of Osteopathic Medicine, Moultrie, USA; 2 Medicine, Lake Erie College of Osteopathic Medicine, Bradenton, USA; 3 Gastroenterology, Borland Groover, Jacksonville, USA

**Keywords:** eosinophilic esophagitis, allergies, atopy, hypersensitivity reactions, dysphagia

## Abstract

Eosinophilic esophagitis (EoE) is an inflammatory condition limited to the esophagus, predominantly consisting of eosinophils, and triggered by hypersensitivity reactions. Recurrent dysphagia secondary to EoE is uncommon in patients with no history of asthma and/or atopic conditions. We are presenting a case of a 48-year-old male suffering from dysphagia for 10 years that worsened over a six-month period. The patient reported no known food and drug allergies, asthma, and other atopic conditions. On esophagogastroduodenoscopy (EGD), the patient fulfilled all five endoscopic reference score (EREFS) criteria, granting a final diagnosis of eosinophilic esophagitis with a score of 6. Biopsy confirmed eosinophilic esophagitis, revealing 20 eosinophils/high-power fields. Skin prick testing was negative. His symptoms had improved at the office follow-up after three weeks after esophageal dilation and proton pump inhibitor (PPI) but did not completely resolve. The patient was then started on fluticasone 440 mcg two times a day. After eight weeks, the patient was symptom-free by taking dual therapy of PPI and fluticasone. The patient was advised to continue taking the daily PPI and then a six-week course of fluticasone if he experienced an exacerbation in his symptoms. In this report, symptom improvement with esophageal dilation, PPI, and fluticasone suggested a successful treatment regimen for EoE in the setting of no known atopy in our patient. Our case highlights EoE in an adult with no known asthma and allergies. The report identifies the importance of a prompt clinical diagnosis and appropriate combination treatment due to the progressive pain and its worsening associated with eosinophilic esophagitis.

## Introduction

Eosinophilic esophagitis (EoE) is an inflammatory disorder limited to the esophagus [1]. The inflammatory infiltrate consists predominantly of eosinophils along with a smaller population of mast cells and basophils [2]. It is hypothesized that this occurs due to a hypersensitivity reaction to certain allergens within foods or the environment [1]. Additionally, it is believed that childhood exposures, genetic predispositions, and atopic diseases may increase the risk of developing EoE [2]. EoE affects approximately 6.6 out of 100,000 and 7.7 out of 100,000 kids and adults, respectively [2]. The prevalence of EoE in the United States is 57/100,000 people [3]. Symptoms include dysphagia, food bolus impaction, regurgitation, angina, and gastroesophageal reflux disease (GERD) [2]. Diagnosis is made clinically by excluding other conditions that may result in similar symptoms. The diagnosis is supported by a biopsy revealing ≥15 eosinophils/high-power field [2]. Although frequently related to hypersensitivity triggers, our case highlights a unique presentation of EoE in an adult with no known history of atopic conditions with successful treatment of symptoms with esophageal dilation, proton pump inhibitor (PPI), and corticosteroid.

## Case presentation

**Figure 1:**
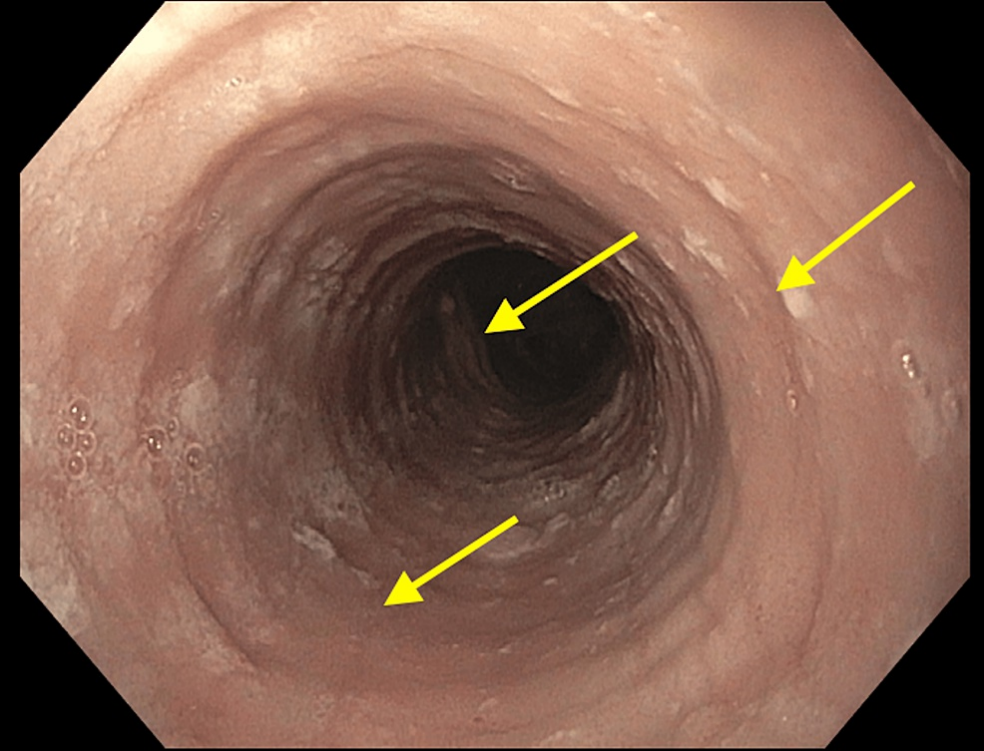
Esophagogastroduodenoscopy showing esophageal rings, strictures, and furrows.

A 48-year-old male presented to the outpatient clinic with symptoms of recurrent dysphagia for 10 years, which progressively worsened over the last six months. The patient reported dysphagia with solids. The patient had no history of impactions and no symptoms of reflux disease. The patient's past medical history was notable for hypertension, which was well controlled with beta-blocker therapy. There was no known personal history of asthma, allergic rhinitis, atopic dermatitis, IgE-mediated food allergies, or environmental allergies, and his family history was benign. The patient reported no alcohol or tobacco use. Vital signs at the clinic showed a blood pressure of 134/82 mmHg, pulse of 64/minute, temperature of 36.7°C, respiratory rate of 19/minute, and O_2_ saturation of 99%. The patient denied nausea, vomiting, hematemesis, hemoptysis, fever, chills, and urinary abnormalities. Physical examination was unremarkable. The patient's weight was stable. Laboratory data including a complete blood count and complete metabolic panel were normal. Esophagogastroduodenoscopy (EGD) was arranged for the evaluation of his symptoms. Esophagogastroduodenoscopy demonstrated esophageal rings, furrows, and strictures (Figure 1).

**Figure 2:**
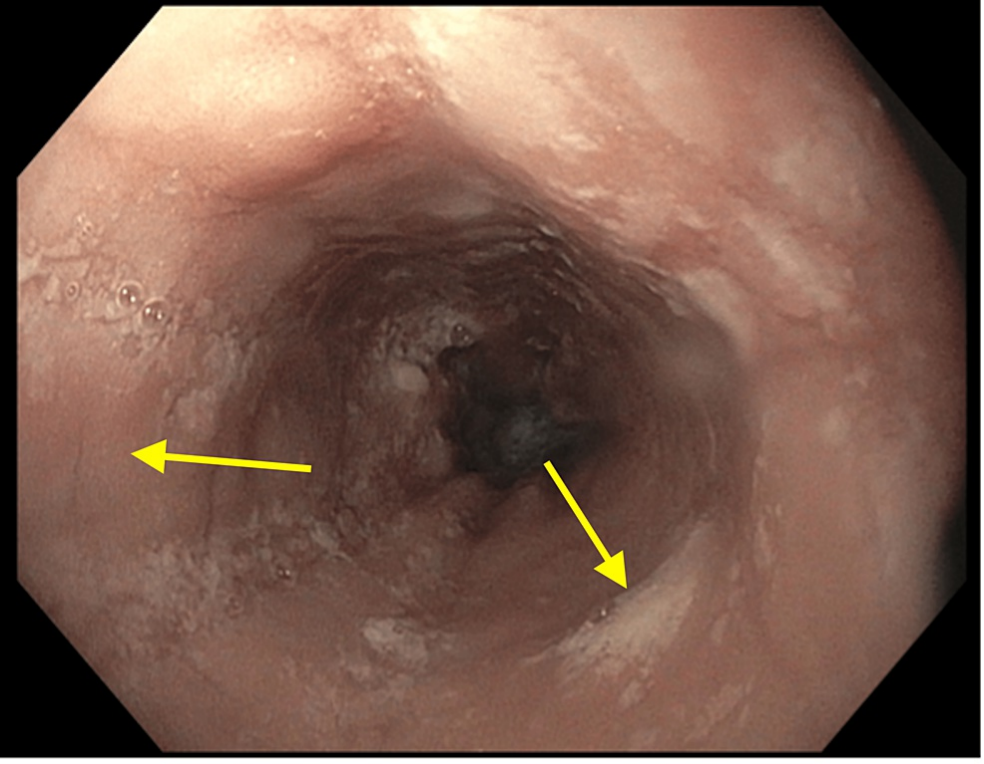
Esophagogastroduodenoscopy showing exudates and mucosal edema.

Additionally, EGD demonstrated mucosal edema and exudates (Figure 2).

Multiple biopsies were obtained. Gentle esophageal dilation was completed with a 56 Fr Maloney dilator. The patient was started on a proton pump inhibitor (PPI) after endoscopy in the setting of mild gastritis and the suggestion of EoE. Pathology report was consistent with EoE, revealing 20 eosinophils/high-power fields. Skin prick testing for allergies was negative. The patient's symptoms had improved at the office follow-up after three weeks, but he reported that his symptoms did not completely resolve. At this point in time, the patient was started on fluticasone 440 mcg twice daily. After eight weeks of both the proton pump inhibitor and fluticasone, the patient's symptoms resolved. The patient was advised to continue using the proton pump inhibitor daily and a short six-week course of fluticasone if he experienced an exacerbation of his symptoms.

## Discussion

The cause of eosinophilic esophagitis is not well understood in adults. Since the first case of adult EoE that was reported in 2002, its incidence has continually increased [4]. The long-term prognosis of EoE is uncertain, but current literature suggests its benign nature other than complications of dysphagia and potential esophageal strictures. The risk factors for EoE include cold or dry climates, the spring and fall seasons due to increased levels of allergens, male gender, and asthma. Although EoE is clinically defined as dysphagia and food impaction in adults, an endoscopic biopsy is required to distinguish it from gastroesophageal reflux disease [5]. Cross-sectional symptom and endoscopy data suggest that some patients with EoE may progress from an inflammatory to a fibrotic process, and this could explain differences in presentation and findings between children and adults with this condition [6]. Additionally, patients with EoE can experience heartburn, chest pain, and odynophagia. The endoscopic reference score (EREFS) was used to help with the diagnosis of EoE in this patient, due to its supported interobserver agreement among practicing and academic gastroenterologists [7]. Current EREFS guidelines outline EoE based on five components: edema, rings, exudates, furrows, and strictures (Table 1) [7].

**Table 1:** Endoscopic reference score (EREFS) for the endoscopic assessment of the esophageal features of eosinophilic esophagitis.

Major Features	Grade
Fixed rings	Grade 0: none; grade 1: mild (subtle circumferential ridges); grade 2: moderate (distinct rings that do not impair the passage of a standard diagnostic endoscope {outer diameter: 8-9.5 mm}); and grade 3: severe (distinct rings that do not permit passage of a diagnostic endoscope)
Exudates	Grade 0: none; grade 1: mild (lesions involving <10% of the esophageal surface area); and grade 2: severe (lesions involving >10% of the esophageal surface area)
Furrows	Grade 0: none; grade 1: mild (vertical lines present without visible depth); and grade 2: severe (vertical lines present with mucosal depth)
Edema	Grade 0: absent (distinct vascularity present); grade 1: mild (the loss of clarity of vascular markings); and grade 2: severe (the absence of vascular markings)
Stricture	Grade 0: absent and grade 1: present

Our patient had a score of 6 on the EREFS criteria (grade 1, rings; grade 1, exudates; grade 1, furrows; grade 2, edema; and grade 1, strictures), granting a differential diagnosis of eosinophilic esophagitis [7]. To further confirm the diagnosis of EoE, the esophageal biopsy report revealed 20 eosinophils/high-power fields. Prompt clinical management was provided due to the patient's increased risk for impaction, regurgitation, and progressive narrowing and scarring to the esophagus. Our report highlights a case of EoE in an adult with no known asthma and atopic conditions. Current literature hypothesizes that the inflammation and fibrostenotic complications of EoE are possible attributors to dysphagia and pain the patients experience [8]. Our patient's symptom improvement with PPI and corticosteroids suggests that the pain and symptoms were related to an inflammatory process. Typical therapy for EoE includes long-term corticosteroids, proton pump inhibitors, and the avoidance of dietary triggers. Experimental treatments, such as biologics, montelukast, and purine analogs, have shown significant reduction in eosinophilic counts. Finally, esophageal dilation is an option when pharmacotherapy is inadequate and dysphagia persists. Our patient showed symptom improvement from esophageal dilation.

One necessary discussion in this case presentation is the pathophysiology of EoE. It is an allergic inflammatory reaction in patients with environmental and genetic risks for atopy. The pathophysiology of EoE is complex and involves various cells and molecules of both adaptive and innate immune pathways. Here, we provide a compact overview of the pathophysiology of EoE. Allergens stimulate the esophageal epithelium, which induces thymic stromal lymphopoietin and interleukin 33, leading to the stimulation of T helper 2 (Th2) cells, natural killer cells, basophils, mast cells, and group 2 innate lymphoid cells [9]. These cells induce interleukin 4, which stimulates Th2 cell differentiation [10]. Interleukin 4 and interleukin 13 induce eotaxin-3, which stimulates eosinophils to secrete interleukin 5 [10]. Eotaxin-3 is the most abundant EoE chemokine and is implicated in eosinophil trafficking to the esophagus [11]. Interleukin 5 promotes eosinophil proliferation, survival, chemotaxis, and activation [12]. Mast cells induce transforming growth factor-beta, which further stimulates eosinophils and fibroblasts. Interleukin 13 downregulates proteins that are important for epithelial integrity and barrier function, resulting in esophageal tissue remodeling, collagen deposition, angiogenesis, and epithelial hyperplasia [13]. This process can account for the dysphagia seen in this patient.

Due to the progressive nature and potential outcomes of untreated EoE, we emphasize the importance of prompt clinical diagnosis and appropriate treatment. Our case presentation is not without limitations. An extensive immunologic and genetic analysis was not performed, which could have revealed an underlying immunodeficiency or Mendelian diseases that might have made this patient more susceptible to EoE. This represents a limitation as it is unknown how the patient's immunologic or genetic risks may have affected his predisposition and progressive development for EoE.

## Conclusions

Eosinophilic esophagitis is a chronic immune-mediated gastrointestinal disorder thought to be related to hypersensitivity reactions. Though the morbidity and mortality of the disease are insignificant, many patients experience dysphagia, food impaction, and GERD, which affect daily living. Diagnosis for EoE has long been related to ruling out other conditions, patient's symptoms, and esophageal biopsy. While eosinophilic esophagitis is not yet completely understood, current treatments have provided some relief, and future medication has shown promise. Despite 10 years of chronic dysphagia and stricture development, our patient's symptoms improved with a combination of esophageal dilation, PPI, and corticosteroids. The patient's improvement of symptoms accentuates the effectiveness of our treatment regimen given, particularly in the setting of no known atopy. This case report highlights an adult case of EoE with a negative skin prick testing that was supported by an endoscopic reference score of 6 and confirmed with a positive esophageal biopsy for EoE. Because the link between non-atopy and EoE still remains unclear, more research is warranted for a clearer understanding of its pathophysiology in order to have pure recognition of EoE and govern the effective treatments and management for patients.
